# Benign Prostatic Hyperplasia and Sexual Dysfunction: Review of the Impact of New Medical and Surgical Therapies on Sexual Health

**DOI:** 10.1007/s11934-025-01279-z

**Published:** 2025-06-09

**Authors:** Danish Singh, Tivoli Nguyen, Patrick Burnam, Jacob Greenberg, Omer Raheem, Wayne Hellstrom

**Affiliations:** 1 Department of Urology, Tulane University School of Medicine, New Orleans, LA #8642, 1430 Tulane Avenue70112 USA; 2 Tulane University School of Medicine, 1430 Tulane Avenue, New Orleans, LA USA; 3 Surgical Subspecialties Institute, Department of Urology, Cleveland Clinic Abu Dhabi, Abu Dhabi, United Arab Emirates

**Keywords:** Sexual dysfunction, Minimally invasive benign prostatic hyperplasia therapy, Ejaculatory disorder, Prostate and sexual function, Retrograde ejaculation, Urination and sexual dysfunction

## Abstract

**Purpose of Review:**

The aim of this review is to evaluate recent literature surrounding the impact of new medical and surgical therapies for benign prostatic hyperplasia (BPH) on sexual health and function.

**Recent Findings:**

Recent randomized controlled trials evaluating patients treated with Optilume, Rezum, Urolift, iTind, and prostatic artery embolism reported minimal associated risks for the development of sexual dysfunction. However, these treatments provide inferior improvement in lower urinary tract symptoms (LUTS) when compared to transurethral resection of the prostate (TURP) and simple prostatectomy. Aquablation demonstrates similar improvement in LUTS while significantly minimizing ejaculatory dysfunction compared to TURP.

**Summary:**

Management of BPH must include patient counseling on the potential risks of sexual dysfunction associated with both medical and surgical therapies. Future research should be guided towards comparing minimally invasive surgical therapies to one another and collecting long-term data on the durability of these therapies on urinary and sexual function.

## Introduction

Benign prostatic hyperplasia (BPH) is a progressive and nonmalignant enlargement of the prostate gland. BPH impacts millions of men worldwide and costs $4 billion annually in healthcare funds in the United States of America (USA) to treat approximately one-third of men over the age of 65 [1, 2]. Clinical sequalae of BPH manifest as significant lower urinary tract symptoms (LUTS), chronic kidney disease, bladder calculi, urinary retention, hematuria, recurrent urinary tract infection, and sexual dysfunction [3]. The relationship between sexual function and BPH has garnered more attention in recent years with the development of minimally invasive BPH therapies. The aim of this review is to evaluate the recent literature surrounding the impact of medical and surgical therapies for BPH on sexual health and function.

## BPH and Erectile Dysfunction

Men with erectile dysfunction (ED) are 1.33–6.24 times more likely to have BPH than the general population [4]. The overall prevalence of ED and LUTS is 70%, and incidence starts to increase at age 40 [5, 6]. In a study of patients with moderate to severe LUTS who were planning to undergo either transurethral or open prostatectomy, LUTS, as determined by the International Prostate Symptom Score (IPSS), was similarly found to be an age-independent risk factor for ED (*P* < 0.001) [7]. Logistic regression analysis observed that men with severe LUTS (IPSS = 20–35) had a 28.7 times greater chance of having ED than men with moderate LUTS (IPSS = 12–19) (*P* < 0.001; odds ratio [OR] 28.65; 95% confidence interval [CI] 14.8–55.5) [7]. The Multinational Survey of the Aging Male (MSAM-7), which surveyed 14,000 men aged 50–80 across the USA and Europe, demonstrated that the presence and severity of LUTS are independent predictors of ED [8]. LUTS severity, measured by the IPSS, was found to significantly predict all subdomains of the International Index of Erectile Function (IIEF), including erectile function, intercourse satisfaction, orgasmic function, sexual desire, and overall satisfaction (*P* < 0.001) [8]. For any clinician treating patients with BPH, it is paramount to screen patients for comorbid urologic conditions due to their high co-occurrence.

## BPH and Ejaculatory Dysfunction

BPH has also been linked to ejaculatory dysfunction (EJD). After adjusting for age and comorbidities, the MSAM-7 noted that moderate or severe LUTS was an independent risk factor for EJD [8]. There was a significant correlation between IPSS and bother scores with MSHQ-EJD scores (*P* < 0.001 and *P* = 0.003, respectively), demonstrating that LUTS severity and bother were strong predictors of EJD [8]. In fact, LUTS was found to be a stronger predictor of EJD than diabetes, hypertension, hyperlipidemia, and heart disease [8]. The Asian Survey of Aging Males similarly reported that the prevalence of EJD increased significantly with LUTS severity and found LUTS to be an independent risk factor, after adjusting for age and comorbidities [9]. Logistic regression on the Danish Prostate Symptom Score–Sex (DAN-PSS-Sex) items found that men with severe LUTS were three times more likely to have EJD than men without LUTS (OR 3.29) [9]. Additionally, men with severe LUTS were almost six times more likely to experience a painful ejaculation (OR 5.95) [9].

## Impact of BPH on Sexual Satisfaction

Researchers reported that severity of BPH/LUTS, measured by the total IPSS score, was associated in patients with depression, defined as a score ≥ 5 on the Patient Health Questionnaire (PHQ)−9 (*P* = 0.046), and somatization, defined as a score ≥ 5 on PHQ-15 (*P* = 0.024) [10]. Although symptom severity was not observed to be correlated with the presence of anxiety, anxious patients were noted to have numerically higher symptom scores than patients without anxiety. However, a clear relationship between BPH with LUTS and psychiatric disorders has yet to be defined [10].

Similarly, a study of 3,500 French men aged 50–80 reported that higher urinary symptom severity predicted a greater degree of sexual dysfunction, as measured by decreases in the frequency of sexual intercourse and the intensity of sexual desire. This influence was independent of patient age [11]. BPH with LUTS also appears to greatly impact a patient’s perception of sexual satisfaction. More specifically, there is a significant negative correlation between sexual satisfaction and LUTS, as a twofold increase in the likelihood of men feeling dissatisfaction with their sexual life was observed with moderate urinary symptoms (IPSS = 9–19) (OR 2.19) and a threefold increase with severe symptoms (IPSS > 19) (OR 3.34), when keeping all other factors equal (age, frequency of sexual relations, comorbidities, previous prostate surgery) [12]. Symptoms of hesitancy, straining, diminished caliber of urinary stream, and wet undergarments were noted to have the most detrimental effects on sexual satisfaction, with all leading to a greater than threefold increase in the odds of sexual dissatisfaction [12]. Data suggest that urinary symptoms secondary to BPH can significantly reduce quality of life and negatively impact sexual health.

## Medical Management of BPH and Sexual Health

### Alpha-Blockers

Alpha-blockers relieve symptoms of LUTS by blocking alpha-1 receptors in the bladder neck and prostatic urethra [13]. A common side effect of alpha-blockers is EJD [14]. Historically, the mechanism of alpha-blocker-mediated ED was postulated to be propagated by relaxation of the bladder neck, resulting in retrograde ejaculation [15, 16]. However, this theory has been challenged by a double-blind, randomized controlled trial (RCT) that revealed no sperm in the post-ejaculate urine of men treated with tamsulosin (Flomax; Boehringer Ingelheim Pharmaceuticals, Ridgefield, Connecticut) [15]. A proposed mechanism is that there are increased receptors of alpha-1a on the seminal vesicles, so treatment with alpha-blockers results in failed contraction of the seminal vesicles and vas deferens [16].

A recent meta-analysis, which included 75 RCTs, revealed that alpha-blockers were significantly associated with EJD [17]. The analysis indicated that more uroselective alpha-blockers had a higher rate of EJD as compared to less uroselective alpha-blockers [17]. Silodosin and tamsulosin have been observed to have the highest rate of EJD, with silodosin associated with the highest odds of EJD across multiple studies [17]. The effect of silodosin on EJD is observed to be dose dependent, with 4 mg twice daily having the higher risk of EJD compared to once daily dosing [17]. A double-blind, crossover RCT, in which 63 healthy volunteers were exposed to silodosin or placebo, revealed reversible azoospermia in patients exposed to an 8 mg daily dose of silodosin, further demonstrating silodosin’s significant impact on ejaculation [18]. This suggests that silodosin may be a viable option for a reversible, non-hormonal male contraceptive [18]. Conversely, no significant risk of EJD has been reported in association with alfuzosin [17].

Another outcome examined by this study was the impact of alpha-blockers on erectile function compared to placebo, and no effect was observed [17]. However, some studies have documented improvement in erectile function with alpha-blocker use. A randomized single-blind study investigated 60 men treated with either tamsulosin or placebo and their self-reported IIEF scores [19]. The study revealed significant improvement in IIEF scores in the tamsulosin group, with 85.7% of patients reporting benefits. In contrast, no significant change in IIEF scores was found in the placebo group, despite the reported emergence of EJD in the tamsulosin group [19]. A study assessing erectile function in patients exposed to alfuzosin therapy versus placebo documented higher scores of erectile function in patients treated with alfuzosin [20]. The impact on erectile function with alpha-blocker therapy may be minimal, given the effect becomes insignificant in a pooled analysis of trials [17].

### 5α-reductase (5 AR) Inhibitors

Dihydrotestosterone (DHT), the primary androgen driving prostate growth, is a key target in the pharmacological treatment of BPH. The two FDA-approved medications targeting DHT, finasteride (Proscar; Merck Pharmaceuticals Rahway, New Jersey) and dutasteride (Avodart; GlaxoSmithKline Brentford, United Kingdom), reduce DHT-driven prostate enlargement by inhibiting the enzyme 5α-reductase (5 AR) and lowering the physiological levels of DHT [19]. This conversion of testosterone to DHT is mediated by two isoforms of 5 AR: type 1, found in various tissues, and type 2, predominantly expressed in the prostate [21]. A recent third isoenzyme was discovered and found to be expressed in castrate-resistant prostate cancer cells, but, for the purposes of BPH, it is clinically insignificant [21]. Dutasteride inhibits both isoforms, whereas finasteride primarily targets type 2 [21]. 5 AR inhibitors are considered first-line treatments of BPH but have been documented as having frequent negative side effects on sexual function, with reductions in libido, ED, and EJD [22–24]. The mechanism of sexual dysfunction is proposed to be a combination of decreased production of nitric oxide in the corpus cavernosum as well as a reduction in DHT levels [22].

Both finasteride and dutasteride have been reported to cause significant changes in sexual function. However, which medication has more sexual side effects remains uncertain due to conflicting data. A meta-analysis of 15 different randomized, placebo-controlled trials of 4,495 subjects revealed a 1.57-fold (95% CI 1.19–2.08) increase in the risk of sexual dysfunction as compared to placebo in both patients receiving 5 AR inhibitors for BPH and androgenic alopecia (AGA) [25]. However, dutasteride did not have a significant increased risk of sexual dysfunction, with a risk ratio of 1.37 (95% CI 0.81–2.32), compared to finasteride, which had a risk ratio of 1.66 (95% CI 1.20–2.30) [25].

A meta-analysis of 17 different randomized, placebo-controlled trials of 17,494 subjects confirmed that rates of sexual dysfunction were significantly increased when using 5 AR inhibitors [24]. In this analysis, dutasteride was noted to have a significantly increased risk of side effects as compared to finasteride, with pooled relative risk of BPH and AGA studies of 2.56 (95% CI 1.48–4.42) [24]. It is apparent there are significant sexual dysfunction side effects from 5 AR inhibitors, but the data do not suggest one 5 AR inhibitor carries a higher relative risk of these side effects compared to the other.

### Phosphodiesterase type 5 (PDE5) Inhibitors

Tadalafil (Cialis; Eli Lilly Indianapolis, Indiana), sildenafil (Viagra; Pfizer New York, New York), and vardenafil (Levitra; Bayer Leverkusen, Germany) are oral phosphodiesterase type 5 (PDE5) inhibitors and have been extensively evaluated for their efficacy in managing BPH-associated ED and LUTS [26–28]. PDE5 inhibitors as monotherapy have demonstrated significant therapeutic value in treating LUTS and ED in BPH patients, with demonstrated improvements in urinary flow and reported erectile function [29].

Although PDE5 inhibitors are effective as monotherapy for treating ED in patients with BPH, their effectiveness in managing both LUTS and enhancing sexual function significantly improves when used as combination therapy [30]. Research has observed that combination therapy with a PDE5 inhibitor and an alpha-blocker provides superior improvement in LUTS and ED than an alpha-blocker alone [29–31]. A meta-analysis conducted in 2014, which included 515 patients from seven different RCTs, observed significant improvements in IIEF scores (*P* = 0.04), IPSS scores (*P* = 0.004), and maximum urinary flow rate (Qmax) values (0.007) in patients managed with PDE5 inhibitors alone and in combination with alpha-blockers [29]. Specifically, when comparing the ED scores of 464 patients (187 managed with PDE5 inhibitor monotherapy and 277 managed with PDE5 inhibitor/alpha-blocker combination therapy), IIEF scores were significantly higher in the combination group than in the monotherapy group (*P* = 0.04), with a combined mean difference of 2.25 and a 95% CI 0.07–4.43 [29]. IPSS scores and Qmax values were also significantly increased in the PDE5 inhibitor/alpha-blocker combination therapy compared to PDE5 inhibitor monotherapy [29]. These results support that PDE5 inhibitor/alpha-blocker combination therapy provide further improvement in both LUTS and ED for male patients suffering from BPH with LUTS and ED.

Studies have observed that a combination of PDE5 inhibitors with 5 AR inhibitors improves urinary symptoms as well as ED symptoms [31]. A study compared the combination therapy of a PDE5 inhibitor and 5 AR inhibitor to PDE5 inhibitor/tamsulosin therapy, examining the impact on urinary flow and ED using IPSS and IIEF scores [31]. It was concluded that while both groups had equivalent improvement in LUTS, the group who received combination therapy of a PDE5 inhibitor and 5 AR inhibitor revealed more significant improvement in sexual function.

## Impact of Surgical Therapies for BPH on Sexual Function

### Transurethral Resection of the Prostate 

Transurethral resection of the prostate (TURP) has long been considered the gold standard for treatment of BPH. Initial studies on the impact of TURP on sexual function offered conflicting data on sexual health sequalae. These historic studies caused controversy within the field regarding this particular impact. However, many of these single-center retrospective studies examined TURP’s impact on sexual health as a secondary outcome and may not have been adequately powered to assess its impact on sexual health [32, 33].

An RCT offered level 2 data on sexual function outcomes between monopolar (M)-TURP and bipolar (B)-TURP [33]. Authors of the study showed no difference in erectile function, sexual desire, orgasmic function, or intercourse satisfaction between M-TURP and B-TURP [33]. A subset of patients reported improved or stable erectile function [33]. However, follow-up RCTs on this subject confirmed no significant difference between ED rates when comparing M-TURP to B-TURP [34, 35]. A larger cohort study examining outcomes of TURPs performed in Switzerland from 2000 to 2005 confirmed that while erections are not influenced by TURP, ejaculatory function was significantly impacted, as DAN-PSS-Sex scores increased from 1.07 to 2.28, *P* < 0.00 [34].

### Transurethral Incision of the Prostate

EJD is a well-known and described sequala of TURP, and, as a result, efforts to create a technique that preserves ejaculatory function have been sought. Initially, it was assumed that bladder neck closure was required to preserve antegrade ejaculation post-TURP. This insight into ejaculatory physiology led to a variation in TURP called transurethral incision of the prostate (TUIP), which involves only incising from the bladder neck to the verumontanum, either unilaterally or bilaterally, while avoiding the resection of prostatic tissue. A prospective RCT showed that TUIP reduced rates of retrograde ejaculation from 52.5% to 22.5% [36]. Bilateral TUIP carries an increased risk of EJD compared to unilateral TUIP (6.7% vs 26.7%, respectively) [37]. Both bilateral and unilateral TUIP continue to demonstrate lower rates of EJD compared to traditional TURP [36, 37]. However, a significant limitation of TUIP is prostatic volume, which is generally < 30 g when this technique is utilized [36].

### Ejaculatory-Preserving TURP

A landmark study published in 1994, using endorectal ultrasonography to identify structures involved in ejaculation, challenged the notion that the bladder neck was vital for the preservation of antegrade ejaculation [38]. This study documented the importance of supramontanal and paracollicular tissues in ejaculation [38]. After this physiologic revelation, techniques termed “Ejaculatory-preserving TURP” were developed and showed varying preservation rates of ejaculation from 80–91%, which was significantly improved from ~ 20% preservation in classic TURP. Ejaculatory-preserving TURPs have not only improved ejaculatory function but have also been noted to improve quality-of-life measures and psychological outcomes [39]. TURP improves LUTS symptoms with minimal impact on erectile function, and ejaculatory-preserving techniques limit the development of EJD (Table 1).

**Table 1.**
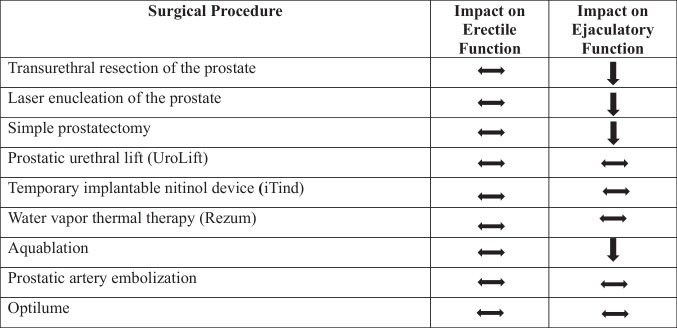
Impact of BPH surgical procedures on sexual function. List of surgical therapies for BPH with their associated impact on erectile and ejaculatory function. Sideways arrow indicates no benefit or no decrease observed in studies, whereas downward arrow indicates a significant reduction in function observed in studies

### Laser Enucleation of the Prostate

Holmium and Thulium laser enucleation of the prostate (HoLEP and ThuLEP, respectively) have gained popularity with the advent of high-powered lasers, allowing urologists to transurethrally enucleate/resect large-sized glands. HoLEP has been investigated the most, with majority of the effort focused on its impact on urinary symptoms. A study conducted in Italy randomized 60 patients between 2002–2003 to receive either HoLEP or TURP and was powered to examine for differences in the change in sexual function using validated questionnaires [40]. These authors found no significant differences in erectile function, intercourse satisfaction, sexual desire, orgasmic function, and overall satisfaction at baseline, 12 months and 24 months [40]. In both groups, however, a significant but equivalent decline in orgasmic function was noted related to EJD, with both procedures at 24 months having a rate of ~ 78.3% retrograde ejaculation [40].

Two retrospective studies, which examined sexual function as a primary outcome post-HoLEP, have reported that while IIEF scores temporarily declined at 6 months post-HoLEP, they either increased to or exceeded baseline levels at the 12-month mark post-HoLEP [31, 41]. Some data suggest that a subgroup of patients undergoing HoLEP, specifically those with normal preoperative erectile function, may experience a decline in erectile function [42]. Significant EJD was also observed in these retrospective cohorts [41–43].

Differences in techniques for laser enucleation of the prostate have been introduced to improve operating time and limit complications. Recently, Thulium lasers have gained interest, as ThuLEP and HoLEP have been compared regarding efficacy and complications [44]. A prospective RCT found no difference in urinary functional outcomes or sexual functional outcomes (measured by changes in IIEF scores) between ThuLEP and HoLEP [44]. A recent RCT reported antegrade ejaculation in enucleating techniques, which preserve tissue at the verumontanum and apex of the prostate, increased the antegrade ejaculation rate from 23% (standard HoLEP) to 77% (ejaculatory-preserving HoLEP) at 12 months post-HoLEP [45]. Top-down HoLEP and standard HoLEP have also been examined in an RCT, which observed no differences in urinary or sexual functional outcomes [46]. Laser enucleation of the prostate improves LUTS symptoms with minimal impact on erectile function, and improvements in technique can reduce the development of EJD post-surgery (Table 1).

### Open Simple Prostatectomy

Open simple prostatectomy (OSP) has remained as one of the gold standard procedures for large prostate glands, with the most data on outcomes [47]. Due to variability in techniques, results from large-scale meta-analyses have been difficult to interpret. A meta-analysis revealed no impact on erectile function from OSP [47]. The PoPAE study comparing OSP to prostatic artery embolization (PAE) reported superior urinary outcomes for the group undergoing OSP and showed no changes to IIEF scores in both groups [48].

Since the advent of laparoscopy, and especially robotics, the technique for OSP has undergone rapid changes. A meta-analysis of trials comparing laparoscopic simple prostatectomy (LSP) and robotic simple prostatectomy (RSP) observed that while RSP had shorter hospital stay, almost all other parameters were equivalent in outcome, including sexual function [49]. Interestingly, in this meta-analysis, the pooled SHIM score improved. While the difference between RSP and LSP was not significant, it did seem to favor the RSP group (mean difference SHIM, 95% CI −1.58, 8.13) [49]. In 1990, the Madigan prostatectomy, which involved sparing the prostatic urethra during enucleation, was described and demonstrated significantly improved ejaculatory function when compared to nonurethral-sparing techniques [50].

A recent prospective study evaluated sexual function outcomes, specifically rates of antegrade ejaculation, by comparing urethral-sparing RSP (Madigan approach) to standard nonurethral-sparing RSP (Millen approach). The study noted significantly higher antegrade ejaculation rates in the urethral-sparing group (81% versus 8.8%), along with significantly improved MSHQ-EJD scores in the urethral-sparing RSP group [51]. Recently, the single-port transvesical RSP has gained popularity, and a recent retrospective study reported equivalent urinary and sexual function outcomes compared to traditional RSP [52]. As with other resection procedures (TURP, HoLEP), the technique for simple prostatectomy has evolved to spare the peri-verumontanum tissue, with subsequent improvements in ejaculatory function observed (Table 1).

## Minimally Invasive Surgical Options

### Prostatic Urethral Lift (UroLift)

Prostatic urethral lift (UroLift; Teleflex Inc., Pleasant, California)) has gained popularity as a minimally invasive surgical option to manage BPH [53]. The purported advantage of the UroLift device is that it can provide compression of prostatic tissue without resection or thermal ablation, thereby minimizing side effects from treatment and leading to faster recovery [53]. Five-year results from the prospective, double-blind RCT, prostatic urethral L.I.F.T study showed durable improvements in IPSS, Quality of Life, and BPH Impact Index scores along with Qmax values [54]. No patients exhibited signs of de novo sexual dysfunction in terms of erectile or ejaculatory function over the 5 years [54].

UroLift was compared to TURP in an RCT, which demonstrated that TURP had superior improvement in IPSS scores and Qmax values, whereas UroLift better preserved ejaculatory function [55]. Another RCT comparing Rezum to UroLift noted similar results, with UroLift showing greater improvement in ejaculatory function and Rezum demonstrating greater improvement in urinary symptoms [56]. Most data has shown that UroLift will minimize ejaculatory and sexual dysfunction post-surgery but at the cost of decreased improvement in LUTS.

### Temporary Implantable Nitinol Device (iTind)

The temporary implantable nitinol device (iTind) procedure (Olympus Surgical Technologies, Westborough, Massachusetts) involves implanting a temporary nitinol device for 5–7 days to create deep incisions at the bladder neck and anterior prostatic fossa [57, 58]. An RCT comparing iTind to sham revealed improvement in IPSS scores and Qmax values but no de novo erectile or ejaculatory function changes [57]. Another RCT comparing iTind to a sham procedure observed no change in erectile or ejaculatory function in patients treated with iTind compared to sham. However, in patients without preoperative ED, there was significant improvement in IIEF scores post-iTind [58].

No RCTs have compared iTind to other BPH surgical procedures. This relatively new BPH procedure needs to be evaluated with long-term data to assess the durability of the procedure in terms of urinary symptoms. In addition, iTind should be compared to other BPH procedures for impact on urinary and sexual function.

### Water Vapor Therapy (Rezum)

Water vapor therapy (Rezum; Boston Scientific, Marlborough, Massachusetts) is a thermal ablative technique used as a minimally invasive surgical option for patients with BPH. Three RCTs have reported that convection water therapy provides durable improvement in IPSS scores and Qmax values, with no patients developing de novo ED or EJD [59–61]. Rezum was also compared to medical therapy using data from two different RCTs, which reported that Rezum did not result in diminished libido, EJD, or ED, while those who underwent medical therapy experienced a negative impact in these aspects of sexual function (Table 1) [62].

### Aquablation

The Aquablation procedure (Procept Biorobotics, San Jose, California) uses a water jet to resect prostatic tissue that has been mapped out by an intraoperative ultrasound, allowing for the sparing of peri-verumontanum tissue [63]. The WATER trial compared Aquablation to the gold standard TURP, with the primary endpoint being a reduction in IPSS scores and secondary endpoints related to sexual function using the MSHQ and IIEF questionnaires [63]. EJD was markedly reduced to 10% in the Aquablation group compared to 36% in the TURP group (Table 1) [63]. Other cohort analyses have confirmed these differences in EJD rates between Aquablation and TURP [64]. A recent prospective study evaluated the impact of Aquablation on circulating tumor cells in men with localized prostate cancer, who were undergoing the procedure [65]. In the study, stable or improved SHIM and MSHQ-ED scores were observed [65]. An interesting avenue for future research would be to investigate how EJD post-Aquablation compares to other minimally invasive BPH surgical therapies.

### PAE

 PAE has gained more popularity as a minimally invasive procedure that relies on intravascular coiling of prostatic arteries, which allows for necrosis of the gland [66]. The PARTEM trial examined the efficacy of PAE versus combined medical therapy with dutasteride/tamsulosin [66]. This trial revealed that IPSS scores and IIEF scores significantly improved with PAE compared to combined medical therapy (Table 1) [66]. A retrospective study also demonstrated that PAE improves urinary symptoms but has no impact on sexual function [67].

During PAE, significant attention has been drawn to coiling of penile collaterals and sexual function, as this is frequently done to prevent nontarget embolization. A retrospective review of 1,007 patients undergoing PAE observed that coiling of penile collaterals had no impact on sexual or ejaculatory function [68]. Again, there is a paucity of data comparing this procedure to other minimally invasive BPH surgical therapies.

### Optilume

The Optilume device (Urotronic Inc., Plymouth, Minnesota), which employs a paclitaxel-coated balloon, is used to create an anterior commissurotomy in the prostate to treat BPH [69]. Recently, in the PINNACLE study, the Optilume device was reported to significantly improve urinary symptoms [69]. Secondary outcomes from the study on sexual function were reported and showed no significant impact on erectile or ejaculatory function (Table 1) [69]. Paucity of data still exists for this treatment due to the recent approval of the device.

## Conclusions

BPH significantly impacts sexual function, with noted associations with ED, EJD, and mental health issues that impact sexual satisfaction. Medical therapy for BPH has long been observed as an effective tool in improving LUTS. However, the side effects of sexual dysfunction associated with these medications are significant and clinically important. Alpha-blockers have been observed to have a significant association with EJD, and 5 AR inhibitors are significantly associated with both ED and EJD. Counseling patients on the associated risk of sexual dysfunction from BPH medical therapy is important, as sexual dysfunction negatively impacts quality of life.

From the patient’s perspective, sexual dysfunction is a crucial factor when considering surgical options for BPH and selecting the most suitable procedure. A survey conducted on 149 men who underwent a BPH procedure within the past two years, along with 150 men seriously considering BPH surgery, revealed that 72% expressed concerns about its impact on sexual function [70]. In addition, when the survey results were stratified by age, a higher proportion of younger males were primarily concerned with sexual-function outcomes [70]. As a result, research is increasingly focusing on the effects of BPH surgical therapies on ED and EJD to address these growing patient concerns.

The importance of the supramontal and paracollicular tissue on preserving antegrade ejaculation was highlighted by Gil-Vernet, which led to the development of “new” ejaculatory-preserving techniques for TURP and simple prostatectomy; these innovations have helped to decrease retrograde ejaculation rates on average by 40% [38, 39]. These anatomical principles continue to guide the development of surgical techniques for new BPH surgical procedures, as evidence by urethral-sparing RSP and Aquablation (purposefully spares apical tissue by adjoining the verumontanum). Minimally invasive surgical therapies reveal significant improvement in LUTS while significantly minimizing ED and EJD as well as decreasing time to full recovery. However, these surgical therapies lack long-term data compared to TURP and simple prostatectomy but do exhibit inferior benefit in LUTS in studies that have compared them to the “gold standard” surgical treatments for BPH.

In conclusion, the current management of BPH must consider not only the relief of urinary symptoms but also the impact on sexual health. A tailored approach to individual patient concerns and the severity of both LUTS and sexual dysfunction is crucial for optimizing both urinary and sexual outcomes. Future research will need to evaluate the long-term sexual health outcomes of new treatments and further refine surgical techniques to balance efficacy in symptom relief with preservation of sexual function.

## Key References

- Ramachandran A, Chavannavar KM, Thiruvengadam G, et al. Ejaculation preserving TURP (Ep-TURP): A viable alternative in sexually active males with BOO—a single-centre prospective randomized study. World J Urol. 2024;42(1):567. doi: 10.1007/s00345-024-05276-8.
  - ⚬ The authors examined ejaculatory-preserving TURP, with a trial specifically focused on ED and EJD as primary outcomes, which is important, given this study specifically evaluated sexual function outcomes.
- Roper C, Slade A, Caras R, et al. Ejaculatory and erectile function outcomes following holmium laser enucleation of the prostate. Prostate. 2024;84(9):791–796. doi: 10.1002/pros.24697.
  - ⚬ This is the most recent study examining large-scale primary ED and EJD outcomes post-HoLEP.
- Porpiglia F, Checcucci E, Amparore D, et al. Urethral-sparing robot-assisted simple prostatectomy: An innovative technique to preserve ejaculatory function overcoming the limitation of the standard Millin approach. Eur Urol. 2021;80(2):222–233. doi: 10.1016/j.eururo.2020.09.028.
  - ⚬ This is the first trial to examine the impact of urethral- preserving RSP compared to standard RSP, with ED and EJD as a primary outcome.
- Elterman D, Shepherd S, Saadat SH, et al. Prostatic urethral lift (UroLift) versus convective water vapor ablation (Rezum) for minimally invasive treatment of BPH: A comparison of improvements and durability in 3-year clinical outcomes. Can J Urol. 2021;28(5):10824–10833.
  - ⚬ This is one of the few RCTs comparing two different minimally invasive BPH surgical techniques, specifically designed to assess differences in ED and EJD.
- Elterman D, Alshak MN, Martinez Diaz S, et al. An evaluation of sexual function in the treatment of lower urinary tract symptoms secondary to benign prostatic hyperplasia in men treated with the temporarily implanted nitinol device. J Endourol. 2023;37(1):74–79. doi: 10.1089/end.2022.0226.
  - ⚬ This iTind trial demonstrated the impact on lower urinary tract function and sexual health. In addition, this is the only RCT on iTind.
- Gilling PJ, Barber N, Bidair M, et al. Five-year outcomes for Aquablation therapy compared to TURP: Results from a double-blind, randomized trial in men with LUTS due to BPH. Can J Urol. 2022;29(1):10960–10968.
  - ⚬ This is the only RCT for minimally invasive BPH surgical therapies that directly compared the surgical therapy to the gold standard TURP.
- Sapoval M, Thiounn N, Descazeaud A, et al. Prostatic artery embolisation versus medical treatment in patients with benign prostatic hyperplasia (PARTEM): A randomised, multicentre, open-label, phase 3, superiority trial. Lancet Reg Health Eur. 2023;31:100672. doi: 10.1016/j.lanepe.2023.100672.
  - ⚬ This is the first trial demonstrating that minimally invasive therapy, specifically PAE, provides equivalent improvement in urinary function as medical therapy while minimizing sexual dysfunction compared to medical therapy for BPH.
- Copelan O, Moss J, Freedman S, et al. Preservation of sexual function with Optilume—a novel treatment for lower urinary tract symptoms secondary to benign prostatic hyperplasia. J Sex Med. 2025; 22(3):446–453. doi: 10.1093/jsxmed/qdae206.
  - ⚬ This is a recent trial examining a new BPH surgical therapy with Optilume and its impact on sexual outcomes.

## Data Availability

No datasets were generated or analysed during the current study.
